# Generating subtour elimination constraints for the TSP from pure integer solutions

**DOI:** 10.1007/s10100-016-0437-8

**Published:** 2016-02-17

**Authors:** Ulrich Pferschy, Rostislav Staněk

**Affiliations:** Department of Statistics and Operations Research, University of Graz, Universitaetsstrasse 15, 8010 Graz, Austria

**Keywords:** Traveling salesman problem, Subtour elimination constraint, ILP solver, Random Euclidean graph, 90C27

## Abstract

The *traveling salesman problem* (*TSP*) is one of the most prominent combinatorial optimization problems. Given a complete graph $G = (V, E)$ and non-negative distances d for every edge, the TSP asks for a shortest tour through all vertices with respect to the distances d. The method of choice for solving the TSP to optimality is a *branch and cut approach*. Usually the *integrality constraints* are relaxed first and all separation processes to identify violated inequalities are done on *fractional solutions*. In our approach we try to exploit the impressive performance of current ILP-solvers and work only with integer solutions without ever interfering with fractional solutions. We stick to a very simple ILP-model and relax the *subtour elimination constraints* only. The resulting problem is solved to integer optimality, violated constraints (which are trivial to find) are added and the process is repeated until a feasible solution is found. In order to speed up the algorithm we pursue several attempts to find as many *relevant* subtours as possible. These attempts are based on the clustering of vertices with additional insights gained from empirical observations and random graph theory. Computational results are performed on test instances taken from the *TSPLIB95* and on *random Euclidean graphs*.

## Introduction

The *Traveling Salesman*/*Salesperson Problem TSP* is one of the best known and most widely investigated combinatorial optimization problems with four famous books entirely devoted to its study (Lawler et al. 1985; Reinelt 1994; Gutin and Punnen 2006; Applegate et al. 2006). Thus, we will refrain from giving extensive references but mainly refer to the treatment in Applegate et al. (2006). Given a complete graph $G = (V, E)$ with $|V| = n$ and $|E| = m=n(n-1)/2$, and nonnegative distances $d_e$ for each $e \in E$, the TSP asks for a shortest tour with respect to the distances $d_e$ containing each vertex exactly once.

Let $\delta (v):=\{e=(v,u)\in E {\mid } u \in V\}$ denote the set of all edges adjacent to $v\in V$. Introducing binary variables $x_e$ for the possible inclusion of any edge $e\in E$ in the tour we get the following classical ILP formulation:

1 $$\begin{aligned}&\hbox {minimize} \quad \sum _{e \in E} d_e x_e \end{aligned}$$

2 $$\begin{aligned}&\quad \quad \hbox {s.t.} \quad \sum _{e \in \delta (v)}{x_e} = 2 \quad \forall \; v \in V, \end{aligned}$$

3 $$\begin{aligned}&\qquad \quad \quad \sum _{\begin{array}{c} {e=(u,v) \in E}\\ {u,v \in S} \end{array}}{x_e} \le |S| - 1 \quad \forall \;S \subset V, S \ne \emptyset , \end{aligned}$$

4 $$\begin{aligned}&\qquad \qquad \qquad \qquad \quad x_e \in \{0, 1\} \quad \forall \; e \in E \end{aligned}$$

Equation (1) defines the *objective function*, (2) is the *degree equation* for each vertex, (3) are the *subtour elimination constraints* (*SEC*), which forbid solutions consisting of several disconnected tours, and finally (4) defines the *integrality constraints*. Note also that some SEC are redundant: For the vertex sets $S \subset V$, $S \ne \emptyset$, and $S^\prime = V \backslash S$ we get pairs of SEC both enforcing the connection of *S* and $S^\prime$.

The established standard approach to solve TSP to optimality, as pursued successfully during the last 30+ years, is a branch-and-cut approach, which solves the LP-relaxation obtained by relaxing the integrality constraints (4) into $x_e \in [0,1]$. In each iteration of the underlying branch-and-bound scheme cutting planes are generated, i.e. constraints that are violated by the current fractional solution, but not necessarily by any feasible integer solution. Since there exists an exponential number of subsets $S \subset V$ implying SEC (3), the computation starts with a small collection of subsets $S \subset V$ (or none at all), and identifies violated SEC as cutting planes in the so-called separation problem. Moreover, a wide range of other cutting plane families were developed in the literature together with heuristic and exact algorithms to find them (see e.g. Applegate et al. 2006; Schrijver 2003, ch. 58). Also the undisputed champion among all TSP codes, the famous *Concorde* package (see Applegate et al. 2006), is based on this principle.

In this paper we introduce and examine another concept for solving the TSP. In Sect. 2 we introduce the basic idea of our approach. Some improvement strategies follow in Sect. 3 with our best approach presented in Sect. 3.4. Since the main contribution of this paper are computational experiments, we discuss them in detail in Sect. 4. The common details of all these tests will be given in Sect. 4.1. In Sect. 5, we present some theoretical results and further empirical observations. Finally, we provide an “Appendix” with illustrations, graphs and two summarizing tables (Tables 5, 6).

## General solution approach

Clearly, the performance of the above branch-and-cut approach depends crucially on the performance of the used LP-solver. Highly efficient LP-solvers have been available for quite some time, but also ILP-solvers have improved rapidly during the last decades and reached an impressive performance. This motivated the idea of a very simple approach for solving TSP without using LP-relaxations explicitly.

The general approach works as follows (see Algorithm 1). First, we relax all SEC (3) from the model and solve the remaining ILP model (corresponding to a *weighted 2-matching problem*). Then we check if the obtained integer solution contains subtours. If not, the solution is an optimal TSP tour. Otherwise, we find all subtours in the integral solution (which can be done by a simple scan) and add the corresponding SEC to the model, each of them represented by the subset of vertices in the corresponding subtour. The resulting enlarged ILP model is solved again to optimality. Iterating this process clearly leads to an optimal TSP tour.
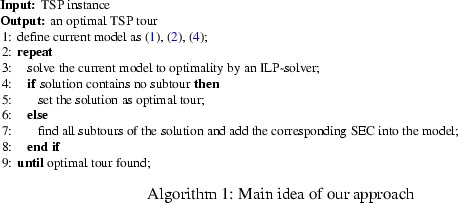


Every execution of the ILP-solver (see line 3) will be called an *iteration*. We define the *set of violated SEC* as the set of all included SEC which were violated in an iteration (see line 7). Figures 3, 6, 7, 8, 9, 10, 11, 12, 13, 14, 15, 16, and 17 in the “Appendix” illustrate a problem instance and the execution of the algorithm on this instance respectively.

It should be pointed out that the main motivation of this framework is its simplicity. The separation of SEC for fractional solutions amounts to the solution of a max-flow or min-cut problem. Based on the procedure by Padberg and Rinaldi (1990), extensive work has been done to construct elaborated algorithms for performing this task efficiently. On the contrary, violated SEC of integer solutions can be found by a simple scan. Moreover, we refrain from using any other additional inequalities known for classical branch-and-cut algorithms, which might also be used to speed up our approach, since we want to underline the strength of modern ILP-solvers in connection with a refined subtour selection process (see Sect. 3.4).

This approach for solving TSP is clearly not new but was available since the earliest ILP formulation going back to Dantzig et al. (1954) and can be seen as folklore nowadays. Several authors followed the concept of generating integer solutions for some kind of relaxation of an ILP formulation and iteratively adding violated integer SEC. However, it seems that the lack of fast ILP-solvers prohibited its direct application in computational studies although it was used in an artistic context by Bosch (2008).

Miliotis (1976) also concentrated on generating integer SEC, but within a fractional LP framework. The classical paper by Crowder and Padberg (1980) applies the iterative generation of integer SEC as a second part of their algorithm after generating fractional cutting planes in the first part to strengthen the LP-relaxation. They report that not more than three iterations of the ILP-solver for the strengthened model were necessary for test instances up to 318 vertices. Also Grötschel and Holland (1991) follow this direction of first improving the LP-model as much as possible, e.g. by running preprocessing, fixing certain variables and strengthening the LP-relaxation by different families of cutting planes, before generating integer subtours as last step to find an optimal tour. It turns out that about half of their test instances never reach this last phase. In contrast, we stick to the pure ILP-formulation without any previous modifications.

From a theoretical perspective, the generation of subtours involves a certain trade-off. For an instance (*G*, *d*) there exists a minimal set of subtours $\mathcal {S}^*$, such that the ILP model with only those SEC implied by $\mathcal {S}^*$ yields an overall feasible, and thus optimal solution. However, in practice we can only find collections of subtours larger than $\mathcal {S}^*$ by adding subtours in every iteration until we reach optimality. Thus, we can either collect as many subtours as possible in each iteration, which may decrease the number of iterations but increases the running time of the ILP-solver because of the larger number of constraints. Or we try to control the number of SEC added to the model by trying to judge their relevance and possibly remove some of them later, which keeps the ILP-model smaller but may increase the number of iterations. In the following we describe various strategies to find the “right” subtours.

### Representation of subtour elimination constraints

The SEC (3) can be expressed equivalently by the following cut constraints:

5 $$\begin{aligned} \sum _{\begin{array}{c} {e=(u,v) \in E}\\ {u \in S, v \not \in S} \end{array}}{x_e} \ge 2 \qquad \forall \;S \subset V, \; S \ne \emptyset \end{aligned}$$

Although mathematically equivalent, the two ways of forbidding a subtour in *S* may result in quite different performances of the ILP-solver. For more details about different ILP-models see Öncan et al. (2009).

It was observed that in general the running time for solving an ILP increases with the number of non-zero entries of the constraint matrix. Hence, we also tested a hybrid variant which chooses between (3) and (5) by picking for each considered set *S* the version with the smaller number of nonnegative coefficients on the left-hand side as follows:

6 $$\begin{aligned} \begin{array}{ll} \displaystyle \mathop {\sum }\limits _{\begin{array}{c} {e=(u,v) \in E}\\ {u,v \in S} \end{array}}{x_e} \ \le \ |S| - 1\\ \displaystyle \mathop {\sum }\limits _{\begin{array}{c} {e=(u,v) \in E}\\ {u \in S, v \not \in S} \end{array}}{x_e} \ \ge \ 2 \end{array}\quad \forall \;\; S \subset V, \; S \ne \emptyset \quad \begin{array}{ll} \hbox {if}&{} |S| \le \displaystyle \frac{2 n + 1}{3}\\ \hbox {if}&{} |S| > \displaystyle \frac{2 n + 1}{3} \end{array} \end{aligned}$$

We performed computational tests of our approach to compare the three representations of SEC, namely (3), (5) and (6), and list the results in Table 1. Technical details about the setup of the experiments can be found in Sect. 4.1.

**Table 1:** Comparison of the behavior of the algorithm for different representations of SEC

Instance	SEC as in (3)			SEC as in (5)			SEC as in (6)		
	t (s)	#iter	#SEC	t (s)	#iter	#SEC	t (s)	#iter	#SEC
kroA150	89	12	82	75	12	82	**62**	**12**	**82**
kroB150	**52**	**13**	**77**	237	13	77	54	13	77
u159	**9**	**5**	**39**	13	5	39	**9**	**5**	**38**
brg180	62	14	56	**36**	**5**	**29**	64	16	67
kroA200	2153	11	95	**1833**	**11**	**95**	2440	11	95
kroB200	45	7	65	146	7	65	**37**	**7**	**65**
tsp225	**149**	**15**	**102**	376	16	105	155	16	106
a280	**114**	**10**	**59**	249	10	56	132	10	63
lin318	7171	13	177	8201	13	177	**7158**	**13**	**177**
gr431	5973	22	186	19,111	22	187	**5925**	**22**	**186**
pcb442	4406	43	215	6186	41	197	**2393**	**43**	**207**
gr666	**33,259**	**14**	**216**	189,421	14	217	40,111	14	216
Mean ratio				2.31	0.95	0.95	0.97	1.02	1.02
RE_A_150	**23**	**12**	**61**	65	12	61	26	12	61
RE_A_200	81	15	84	139	15	84	**76**	**15**	**84**
RE_A_250	156	14	82	208	14	82	**133**	**14**	**82**
RE_A_300	**534**	**14**	**123**	4819	14	123	692	14	123
RE_A_350	**404**	**9**	**110**	789	9	110	650	9	110
RE_A_400	49,234	16	179	247,511	16	179	**24,619**	**16**	**179**
RE_A_450	4666	8	117	13,806	8	117	**3022**	**8**	**117**
RE_A_500	68,215	12	167	155,977	12	167	**30,809**	**12**	**167**
Mean ratio				3.39	1.00	1.00	0.93	1.00	1.00
Mean ratio all				2.74	0.97	0.97	0.95	1.01	1.01

*Mean ratios* refer to the arithmetic means over ratios between t (s)/#iter./#SEC for the approaches using the SEC represented as in (5) and (6) respectively and t (s)/#iter./#SEC for the approach using the SEC represented as in (3). “t (s)” is the time in seconds, “#iter” the number of iterations and “#SEC” the number of SEC added to the ILP before starting the last iteration

The data for the best approach with respect to running time is given in bold

It turned out that the three versions sometimes (but not always) lead to huge differences in running time (up to a factor of 5). This is an interesting experience that should be taken into consideration also in other computational studies. From our limited experiments it could be seen that version (5) was inferior most of the times (with sometimes huge deviations) whereas only a small dominance of the hybrid variant (6) in comparison with the standard version (3) could be observed. This is due to the small size of most subtours occurring during the solution process (the representation (3) equals to the representation (6) in these cases). But since also bigger subtours can occur (mostly in the last iterations), we use the representation (6) for all further computational tests.

## Generation of subtours

As pointed out above, the focus of our attention lies in the generation and selection of a “good” set of SEC, including as many as possible of those required by the ILP-solver to determine an optimal solution which is also feasible for TSP, but as few as possible of all others which only slow down the performance of the ILP-solver.

Trying to strike a balance between these two goals we followed several directions, some of them motivated by theoretical results, others by visually studying plots of all subtours generated during the execution of Algorithm 1.

### Subtour elimination constraints from suboptimal integer solutions

Many ILP-solvers report all feasible integer solutions found during the underlying branch-and-bound process. In this case, we can also add all corresponding SEC to the model. These constraints can be considered simply as part of the set of violated SEC. Not surprisingly, these additional constraints always lead to a decrease in the number of iterations for the overall computation and to an increase in the total number of SEC generated before reaching optimality (see Table 2). While the time consumed in each iteration is likely to increase, it can also be observed that the overall running time is often decreased significantly by adding all detected subtours to the model. On the other hand, for the smaller number of instances where this is not the case, only relatively modest increases of running times are incurred. Therefore, we stick to adding all detected SEC for the remainder of the paper. The algorithm in this form will be called *BasicIntegerTSP*.

**Table 2:** Using all constraints generated from all feasible solutions found during the solving process versus using only the constraints generated from the final ILP solutions of each iteration

Instance	Only subtours from ILP-optima			All subtours: BasicIntegerTSP		
	t (s)	#iter	#SEC	t (s)	#iter	#SEC
kroA150	62	12	82	**19**	**7**	**136**
kroB150	**54**	**13**	**77**	179	8	148
u159	9	5	38	**6**	**4**	**49**
brg180	64	16	67	**44**	**4**	**103**
kroA200	2440	11	95	**677**	**8**	**237**
kroB200	37	7	65	**31**	**5**	**121**
tsp225	**155**	**16**	**106**	178	9	261
a280	**132**	**10**	**63**	157	11	143
lin318	7158	13	177	**6885**	**8**	**357**
gr431	5925	22	186	**2239**	**9**	**453**
pcb442	**2393**	**43**	**207**	2737	11	501
gr666	40,111	14	216	**17,711**	**8**	**789**
Mean ratio				0.95	0.60	2.17
RE_A_150	26	12	61	**23**	**8**	**100**
RE_A_200	76	15	84	**72**	**7**	**163**
RE_A_250	**133**	**14**	**82**	138	9	186
RE_A_300	**692**	**14**	**123**	866	6	295
RE_A_350	650	9	110	**411**	**5**	**252**
RE_A_400	24,619	16	179	**8456**	**8**	**454**
RE_A_450	3022	8	117	**2107**	**5**	**279**
RE_A_500	30,809	12	167	**15,330**	**6**	**436**
Mean ratio				0.79	0.55	2.26
Mean ratio all				0.88	0.58	2.20

*Mean ratios* refer to the arithmetic means over ratios between t (s)/#iter./#SEC for BasicIntegerTSP over t (s)/#iter./#SEC for the other approach. “t (s)” is the time in seconds, “#iter” the number of iterations and “#SEC” the number of SEC added to the ILP before starting the last iteration

The data for the best approach with respect to running time is given in bold

### Clustering into subproblems

It can be observed that many subtours have a local context, meaning that a small subset of vertices separated from the remaining vertices by a reasonably large distance will always be connected by one or more subtours, independently from the size of the remaining graph (see also Figs. 3, 6, 7, 8, 9, 10, 11, 12, 13, 14, 15, 16, and 17 in the “Appendix”). Thus, we aim to identify *clusters* of vertices and run the BasicIntegerTSP on the induced subgraphs with the aim of generating within a very small running time the same subtours occurring in the execution of the approach on the full graph. Furthermore, we can use the optimal tour from every cluster to generate a corresponding SEC for the original instance and thus enforce a connection to the remainder of the graph.

For our purposes the clustering algorithm should fulfill the following properties:

- *Clustering quality* The obtained clusters should correspond well to the distance structure of the given graph, as in a classical geographic clustering.
- *Running time* Should be low relative to the running time required for the main part of the algorithm.
- *Cluster size* If clusters are too large, solving the TSP takes too much time. If clusters are too small, only few SEC are generated.

Clearly, there is a huge body of literature on clustering algorithms (see e.g. Jain and Dubes 1988) and selecting one for a given application will never satisfy all our objectives. Our main restriction was the requirement of using a clustering algorithm which works also if the vertices are not embeddable in Euclidean space, i.e. only arbitrary edge distances are given. Simplicity being another goal, we settled for the following approach described in Algorithm 2:
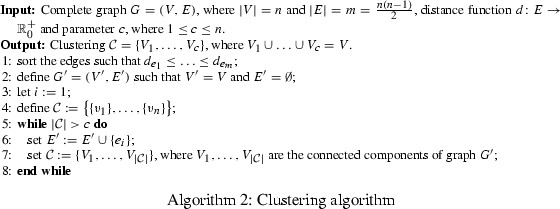


First, we fix the number of clusters *c* with $1 \le c \le n$ and sort the edges in increasing order of distances (see line 1). Then we start with the empty graph $G^\prime = (V^\prime , E^\prime )$ (line 2) containing only isolated vertices (i.e. *n* clusters) and add iteratively edges in increasing order of distances until the desired number of clusters *c* is reached (see lines 5 and 6). In each iteration the current clustering is implied by the connected components of the current graph (see line 7). We denote this *clustering approach* by $C {\mid } c$. Note that this clustering algorithm does not make any assumptions about the underlying TSP instance and does not exploit any structural properties of the *Metric TSP* or the *Euclidean TSP*.

It was observed in our computational experiments that the performance of the TSP algorithm is not very sensitive to small changes of the cluster number *c* and thus a rough estimation of *c* is sufficient. The behavior of the running time as a function of *c* can be found for particular test instances in Fig. 19, see Sect. 4.2 for further discussion.

### Restricted clustering

Although the clustering algorithm (see Algorithm 2) decreases the computational time of the whole solution process for some test instances, we observed a certain shortcoming. There may easily occur clusters consisting of isolated points or containing only two vertices. Clearly, these clusters do not contribute any subtour on their own. Moreover, the degree constraints (2) guarantee that each such vertex is connected to the remainder of the graph in any case. The connection of these vertices to some “neighboring” cluster enforced in BasicIntegerTSP implies that the clustering yields different subtours for these neighbors and not the violated SEC arising in BasicIntegerTSP.

To avoid this situation, we want to impose a minimum cluster size of 3. An easy way to do so is as follows: After reaching the *c* clusters, continue to add edges in increasing order of distances (as before), but add an edge only, if it is incident to one of the vertices in a connected component (i.e. cluster) of size one or two. This means basically that we simply merge these small clusters to their nearest neighbor with respect to the actual clustering. Note that this is a step-by-step process and it can happen that two clusters of size 1 merge first before merging the resulting pair to its nearest neighboring cluster. The resulting *restricted clustering approach* will be denoted by $RC_3 {\mid } c$.

Against our expectations, the computational experiments (see Sect. 4) show that this approach often impacts the algorithm in the opposite way (see also Fig. 19, Table 6 in the “Appendix”) if compared for the same original cluster size *c*.

Surprisingly, we could observe an interesting behavior if $c \approx n$. In this case, the main clustering algorithm (see Algorithm 2) has almost no effect, but the “post-phase” which enforces the minimum cluster size yields a different clustering on its own. This variant often beats the previous standard clustering algorithm with $c \ll n$ (see Table 6 in the “Appendix”). Note that we cannot fix the actual number of clusters $c^\prime$ in this case. But our computational results show that $c^\prime \approx \frac{n}{5}$ usually holds if the points are distributed relatively uniformly in the Euclidean plane and if the distances correspond to their relative Euclidean distances (see Fig. 18 in the “Appendix”).

### Hierarchical clustering

It was pointed out in Sect. 3.2 that the number of clusters *c* is chosen as an input parameter. The computational experiments in Sect. 4.2 give some indication on the behavior of Algorithm 2 for different values of *c*, but fail to provide a clear guideline for the selection of *c*. Moreover, from graphical inspection of test instances, we got the impression that a larger number of relevant SEC might be obtained by considering more clusters of moderate size. In the following we present an idea that takes both of these aspects into account.

**Fig. 1:**
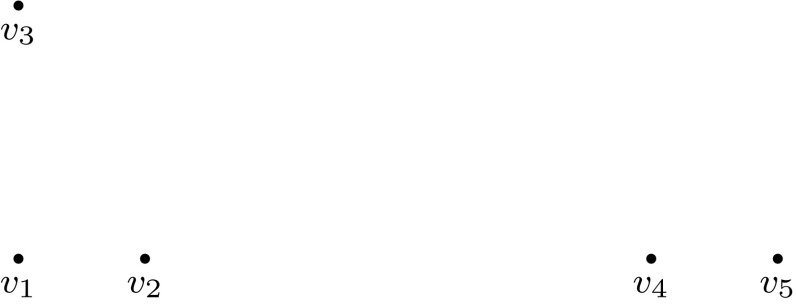
Example illustrating the hierarchical clustering: vertices of the TSP instance. Distances between every two vertices correspond to their respective Euclidean distances in this example

**Fig. 2:**
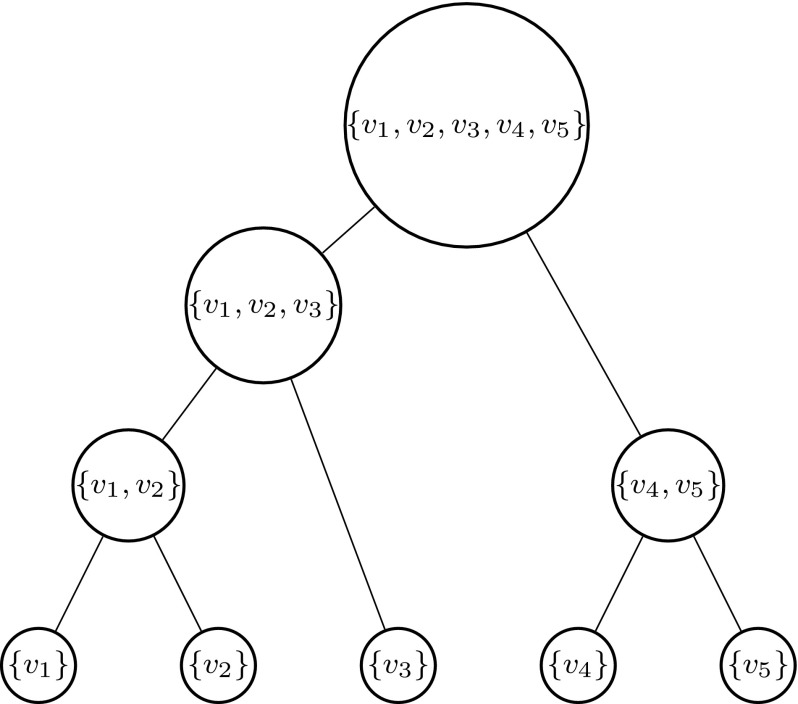
Example illustrating the hierarchical clustering: clustering tree

**Fig. 3:**
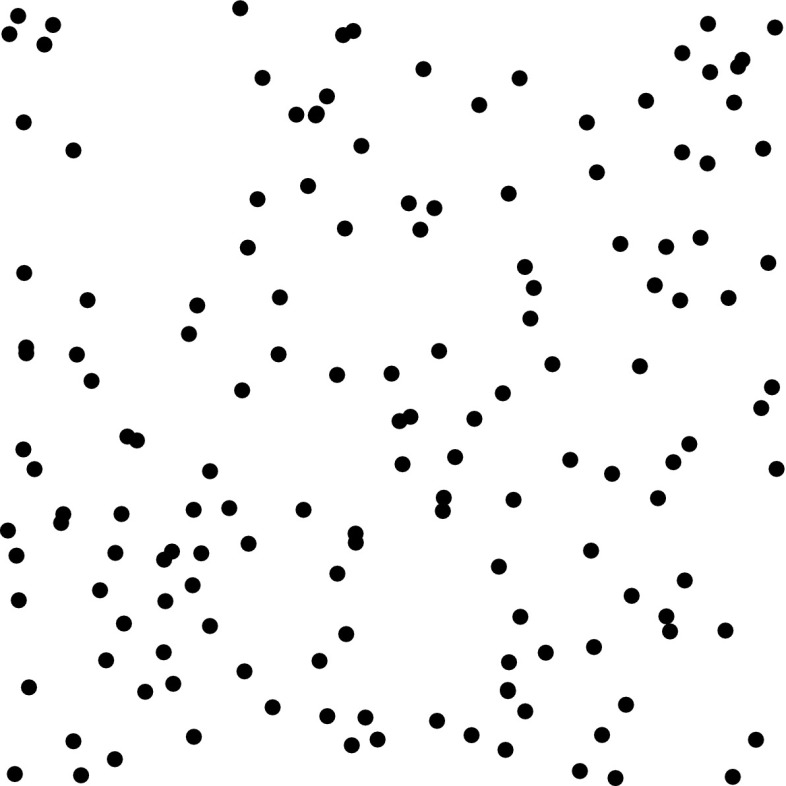
Instance *RE_A_150*. Euclidean distances between vertices

In our *hierarchical clustering* process denoted by *HC* we do not set a cluster number *c*, but let the clustering algorithm continue until all vertices are connected (this corresponds to $c=1$). The resulting clustering process can be represented by a binary *clustering tree* which is constructed in a bottom-up way. The leaves of the tree represent isolated vertices, i.e. the *n* trivial clusters given at the beginning of the clustering algorithm. Whenever two clusters are merged by the addition of an edge, the two corresponding tree vertices are connected to a new common parent vertex in the tree representing the new cluster. At the end of this process we reach the root of the clustering tree corresponding to the complete vertex set. An example of such a clustering tree is shown in Figs. 1 and 2.

Now, we go through the tree in a bottom-up fashion from the leaves to the root. In each tree vertex we solve the TSP for the associated cluster, after both of its child vertices were resolved. The crucial aspect of our procedure is the following: All SEC generated during such a TSP solution for a certain cluster are propagated and added to the ILP model used for solving the TSP instance of its parent cluster. Obviously, at the root vertex the full TSP is solved.

The advantage of this strategy is the step-by-step construction of the violated SEC. A disadvantage is that many constraints can make sense in the local context but not in the global one and thus too many constraints could be generated in this way. Naturally, one pays for the additional SEC by an increase in computation time required to solve a large number of—mostly small—TSP instances. To avoid the solution of TSPs of the same order of magnitude as the original instance, it makes sense to impose an upper bound *u* on the maximum cluster size. This means that the clustering tree is partitioned into several subtrees by removing all tree vertices corresponding to clusters of size greater than *u*. After resolving all these subtrees we collect all generated SEC and add them to the ILP model for the originally given TSP. This approach will be denoted as $HC {\mid } u$. Computational experiments with various choices of *u* indicated that $u = 4 \frac{n}{\log _2{n}}$ would be a good upper bound.

Let us take a closer look at the problem of including too many SEC which are redundant in the global graph context. Of course the theoretical “best” way would be to check which of the propagated SEC were not used during the runs of the ILP solver and drop them. To do this, it would be necessary to get this information from the ILP solver which often is not possible.

However, we can try to approximately identify subtours which are not only locally relevant in the following way: All SEC generated in a certain tree vertex, i.e. for a certain cluster, are marked as *considered SEC*. Then we solve the TSP for the cluster of its parent vertex in the tree without using the subtours marked as *considered*. If we generate such a considered subtour again during the solution of the parent vertex, we take this as an indicator of global significance and add the constraint permanently for all following supersets of this cluster. If we set the upper bound *u*, we take also all SEC found in the biggest solved clusters. This approach will be denoted as $\textit{HCD} {\mid } u$.

Of course, it is only a heuristic rule and one can easily find examples, where this prediction on a subtour’s relevance fails, but our experiments indicate that $\textit{HCD} {\mid } 4 n / \log _2{n}$ is the best approach we considered. A comparison with other hierarchical clustering methods for all test instances can be found in Table 5 in the “Appendix”. It can be seen that without an upper bound we are often not able to find the solution at all (under time and memory constraints we made on the computational experiments). In the third and fourth column we can see a comparison between approaches both using the upper bound $u = 4 \frac{n}{\log _2{n}}$ where the former collects all detected SEC and the latter allows to drop those which seem to be relevant only in a local context. Both these methods beat BasicIntegerTSP (for the comparison of this approach with other presented algorithms see the computational experiments in Sect. 4).

## Computational experiments

In the following the computational experiments and their results will be discussed. Additional illustrative material can be found in an accompanying technical report, Pferschy and Staněk (2015), which is an extended version of this paper.

### Setup of the computational experiments

All tests were run on an *Intel(R) Core(TM) i5-3470 CPU @ 3.20GHz with 16 GB RAM* under *Linux* 1 and all programs were implemented in *C++* 2 by using the *SCIP* MIP-solver^3^ (see Achterberg 2009) together with *CPLEX* as LP-solver. It has often been discussed in the literature (see e.g. Naddef and Thienel 2002) and in personal communications that ILP-solvers are relatively unrobust and often show high variations in their running time performance, even if the same instance is repeatedly run on the same hardware and same software environment. Our first test runs also exhibited deviations up to a factor of 2 when identical tests were repeated. Thus we took special care to guarantee the relative reproducibility of the computational experiments: No additional swap memory was made available during the tests, only one thread was used and no other parallel user processes were allowed. This leads to a high degree of reproducibility in our experiments. However, this issue makes a comparison to other simple approaches, which were tested on other computers under other hardware and software conditions, extremely difficult.

We used two groups of test instances: The first group is taken from the well-known TSPLIB95 (Reinelt 1995), which contains the established benchmarks for TSP and related problems. From the collection of instances we chose all those with (i) at least 150 and at most 1000 vertices and (ii) which could be solved in at most 12 hours by our BasicIntegerTSP. It turned out that 25 instances of the TSPLIB95 fall into this category (see Table 6), the largest having 783 vertices.

We also observed some drawbacks of these instances: Most of them (23 out of 25) are defined as point sets in the Euclidean plane with distances corresponding to the Euclidean metric or as a set of geographical cities, i.e. points on a sphere. Moreover, they often contain substructures like meshes or sets of colinear points and finally, since all distances are rounded to the nearest integer, there are many instances which have multiple optimal solutions. These instances are relatively unstable with respect to solution time, number of iterations, and—important for our approach—cardinality of the set of violated SEC. For our approach instances with a mesh geometry (e.g. *ts225* from TSPLIB95) were especially prone to unstable behavior, such as widely varying running times for minor changes in the parameter setting. This seems to be due to the fact that these instances contain many 2-matchings with the same objective function value, and thus the search process for a feasible TSP tour can vary widely (for more details see Pferschy and Staněk 2015).

In order to provide further comparisons, we also defined a set of instances based on *random Euclidean graphs*: In a unit square $[0, 1]^2$ we chose *n* uniformly distributed points and defined the distance between every two vertices as their respective Euclidean distance.^4^ These *random Euclidean instances* eliminate the potential influence of substructures and always have only one unique optimal solution in all stages of the solving process. We created 40 such instances named *RE_X_n* where $n \in \{150, 200, 250, \ldots , 500\}$ indicates the number of vertices and $X \in \{A, B, C, D, E\}$.

The running times of our test instances, most of them containing between 150 and 500 vertices, were often within several hours. Since we tested many different variants and configurations of our approach, we selected a subset of these test instances to get faster answers for determining the best algorithm settings for use in the final tests. This subset contains 12 (of the 25) TSPLIB instances and one random instances for every number of vertices *n* (see e.g. Table 1).

All our running time tables report the name of the instance, the running time (**t (s)**) in wall-clock seconds (rounded down to nearest integers), the number of iterations (**#iter**), i.e. the number of calls to the ILP-solver in the main part of our algorithm (without the TSP solutions for the clusters) and the number of SEC (**#SEC**) added to the ILP model in the last iteration, i.e. the number of constraints of the model which yielded an optimal TSP solution. We often compare two approaches in a table by taking the **mean ratio**, i.e. we compute the quotients between the particular columns t (s)/#iter/#SEC of the compared method over the first “reference” method on the same instances and then we report the arithmetic mean of these quotients.

### Computational details for selected examples

Let us now take a closer look at two instances in detail. While this serves only as an illustration, we studied lots of these special case scenarios visually during the development of the clustering approach to gain a better insight into the structure of subtours generated by BasicIntegerTSP.

**Fig. 4:**
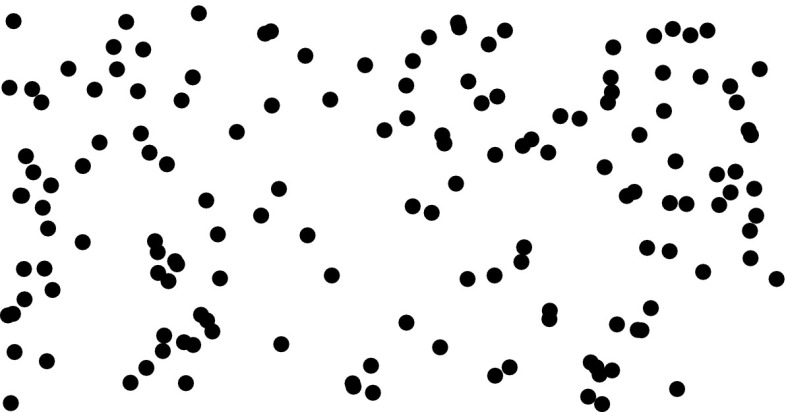
Instance *kroB150*. Euclidean distances between vertices

**Fig. 5:**
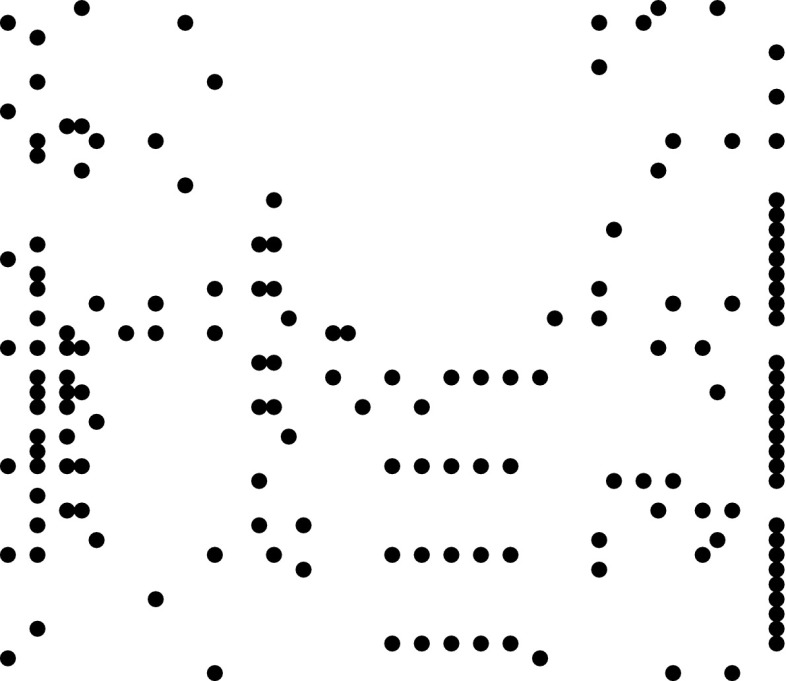
Instance *u159*. Euclidean distances between vertices

**Fig. 6:**
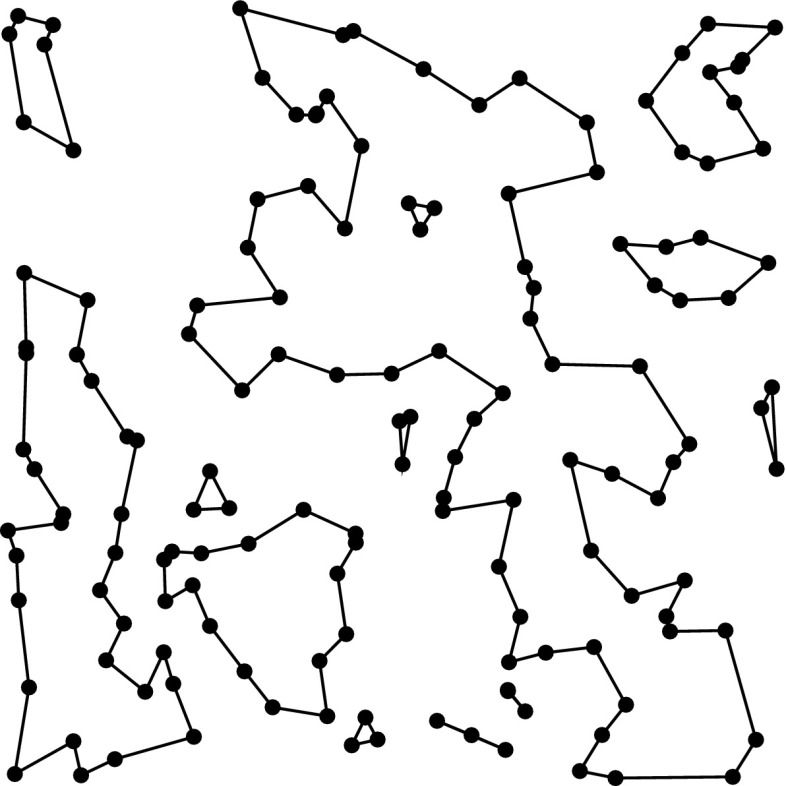
Instance *RE_A_150*: Main idea of our approach—iteration 0

**Fig. 7:**
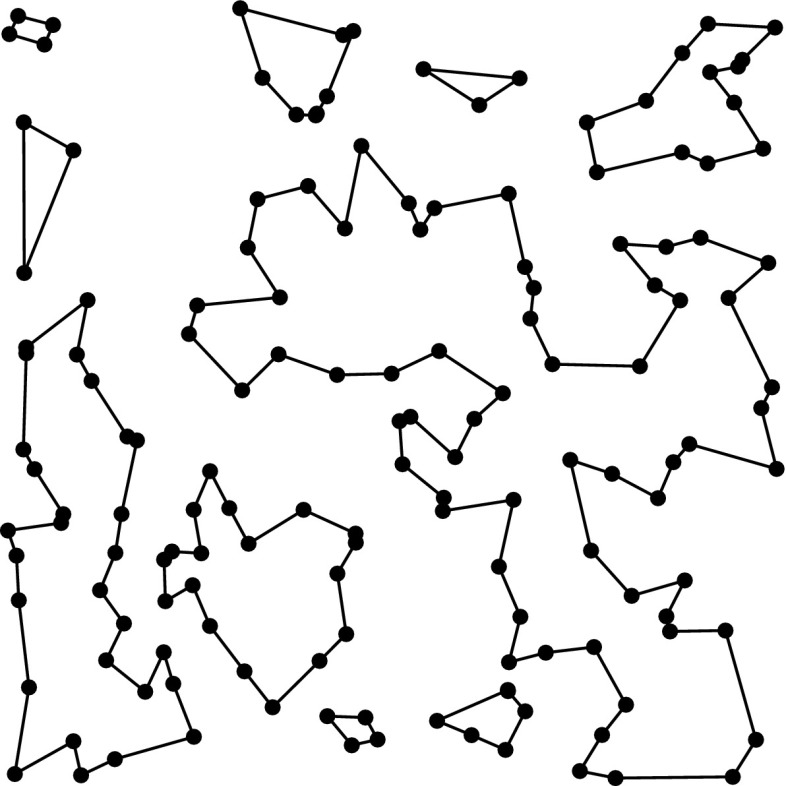
*RE_A_150*: iteration 1

**Fig. 8:**
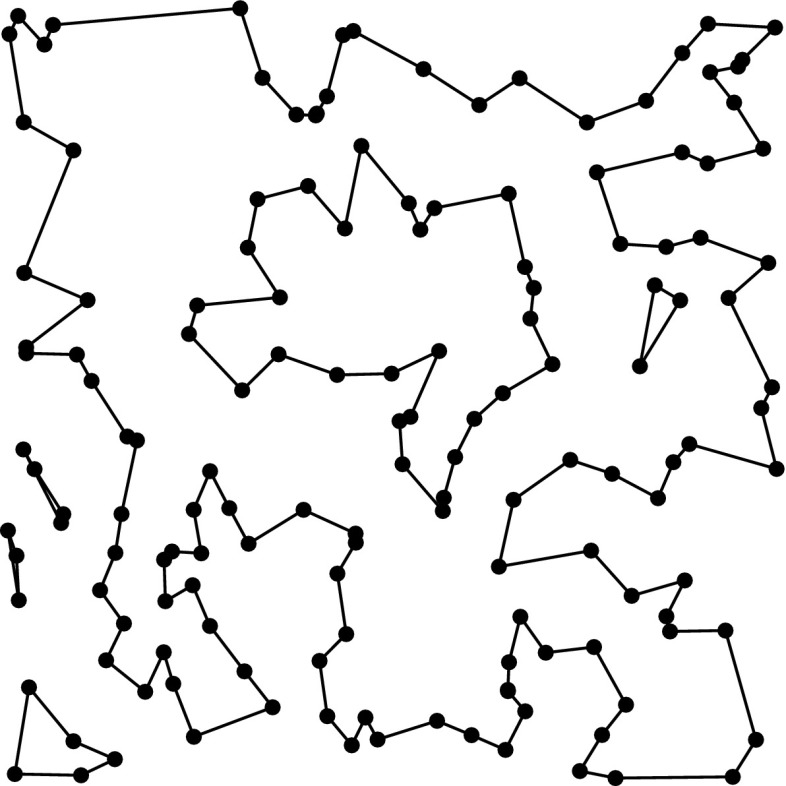
*RE_A_150*: iteration 2

**Fig. 9:**
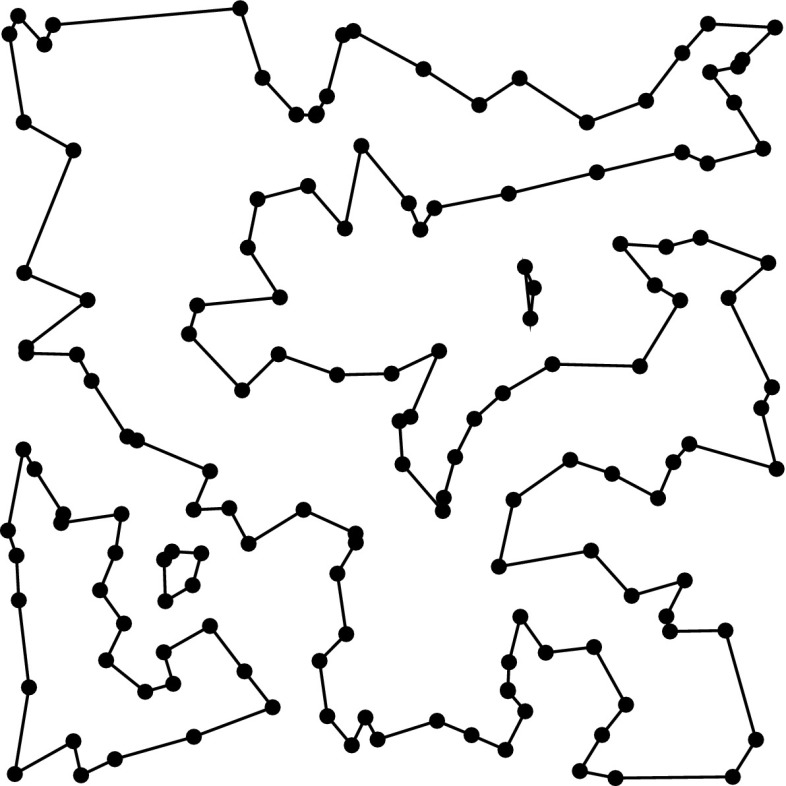
*RE_A_150*: iteration 3

**Fig. 10:**
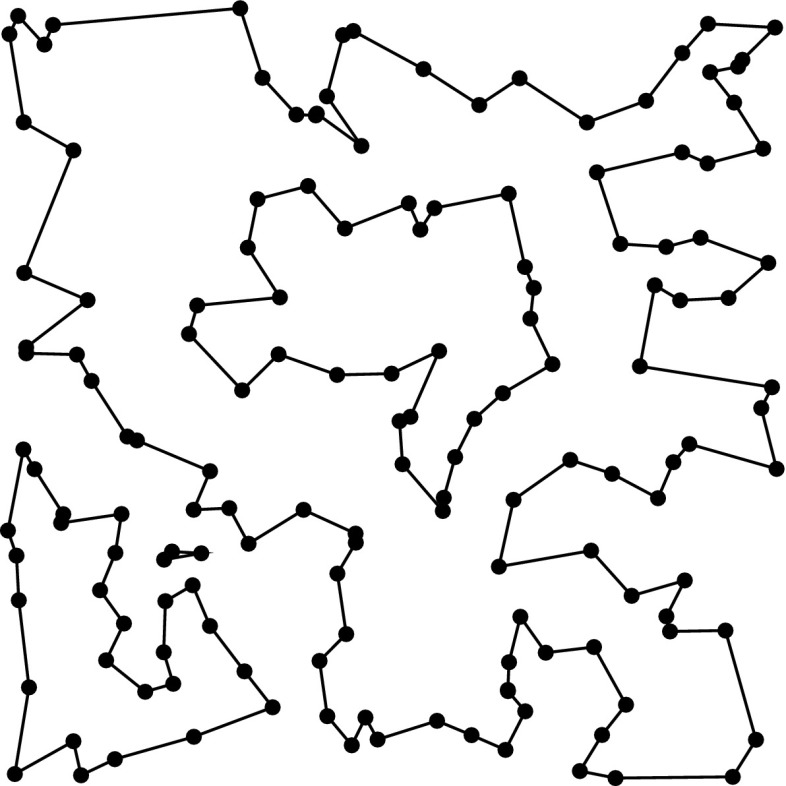
*RE_A_150*: iteration 4

**Fig. 11:**
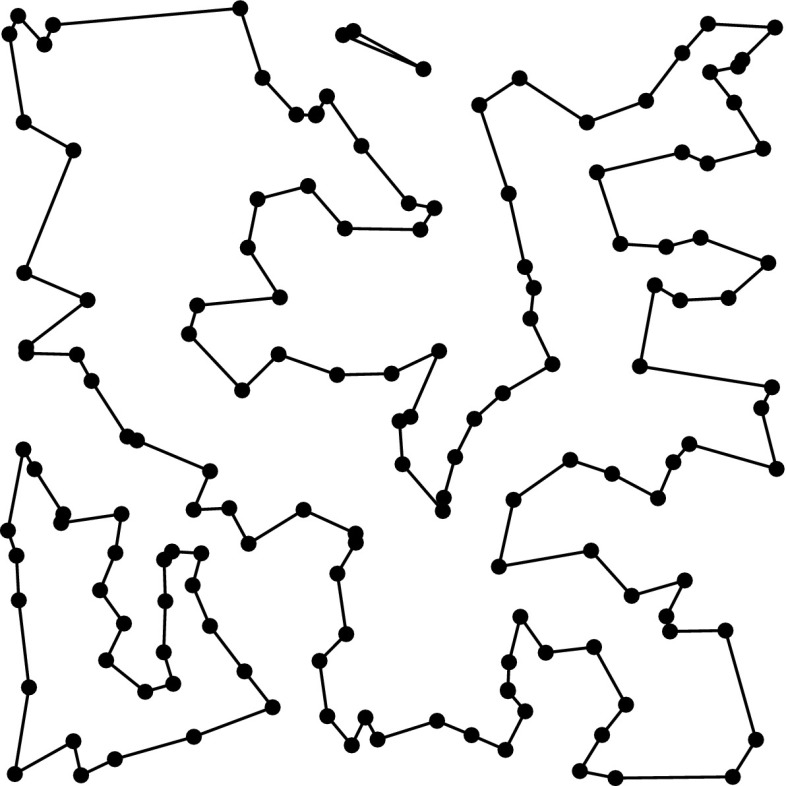
*RE_A_150*: iteration 5

**Fig. 12:**
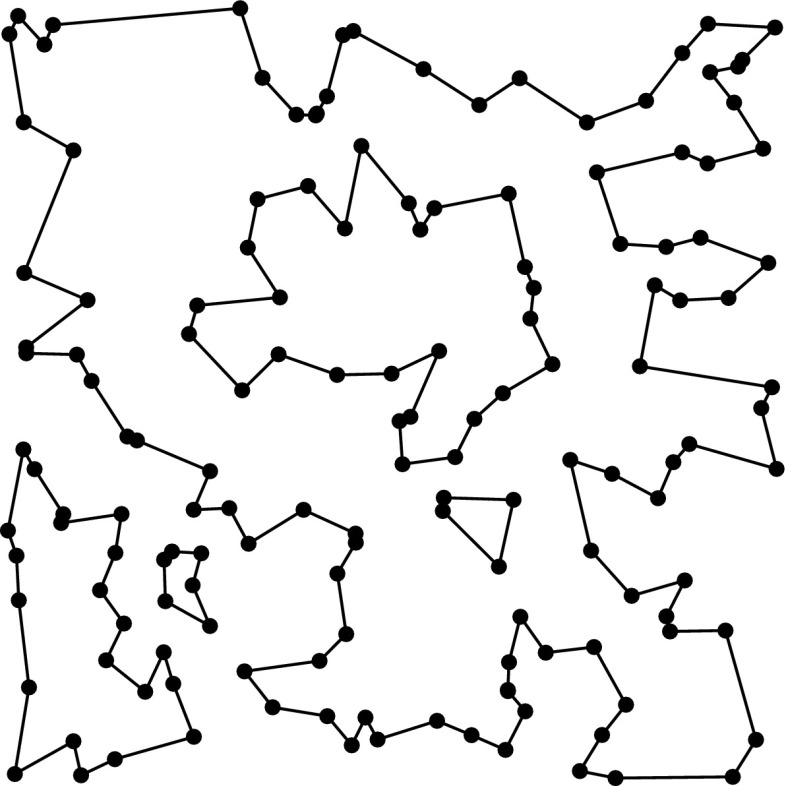
*RE_A_150*: iteration 6

**Fig. 13:**
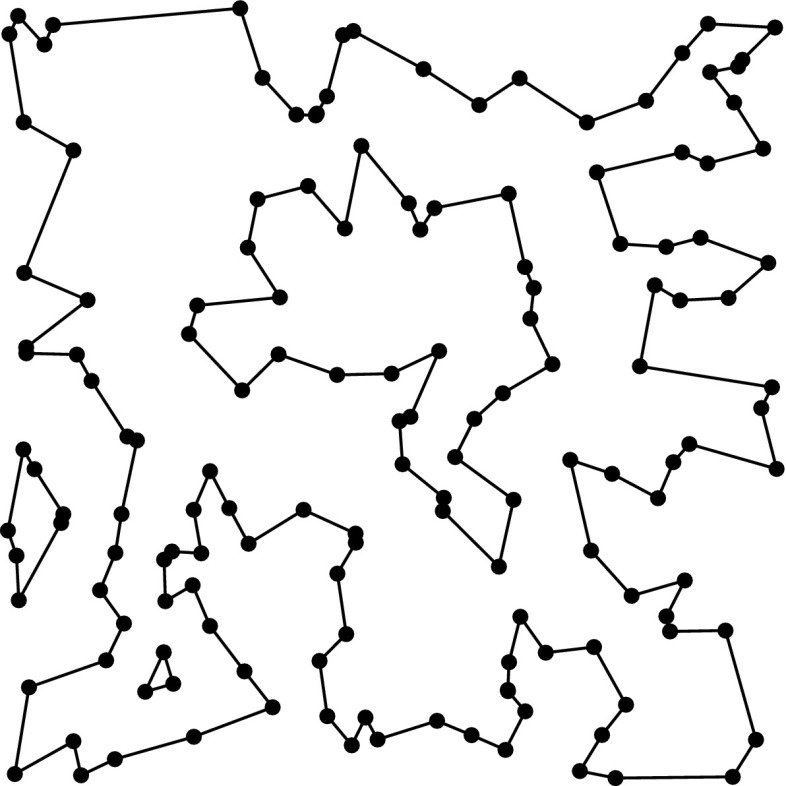
*RE_A_150*: iteration 7

**Fig. 14:**
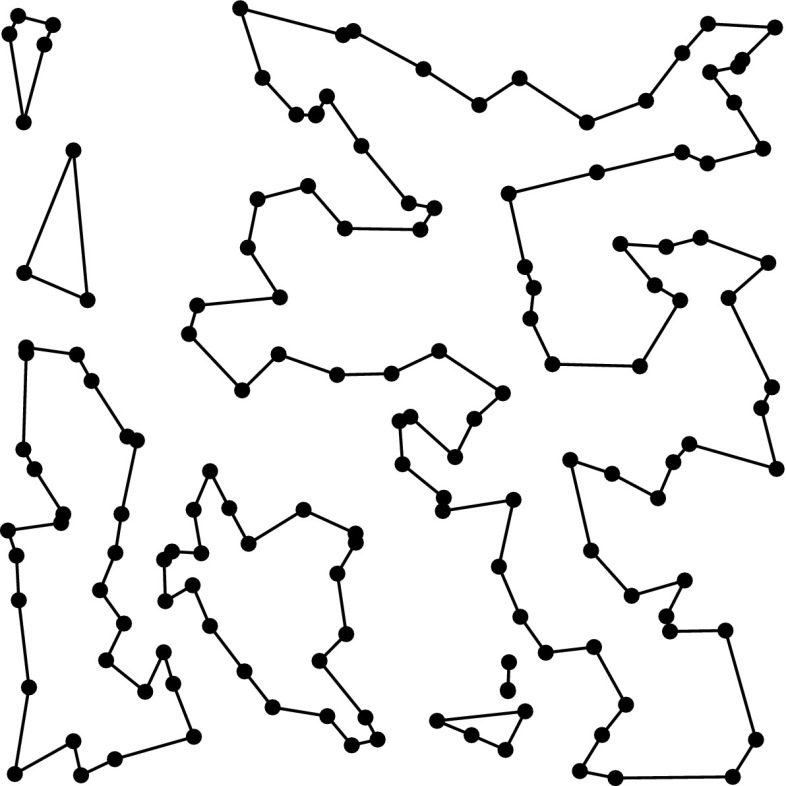
*RE_A_150*: iteration 8

**Fig. 15:**
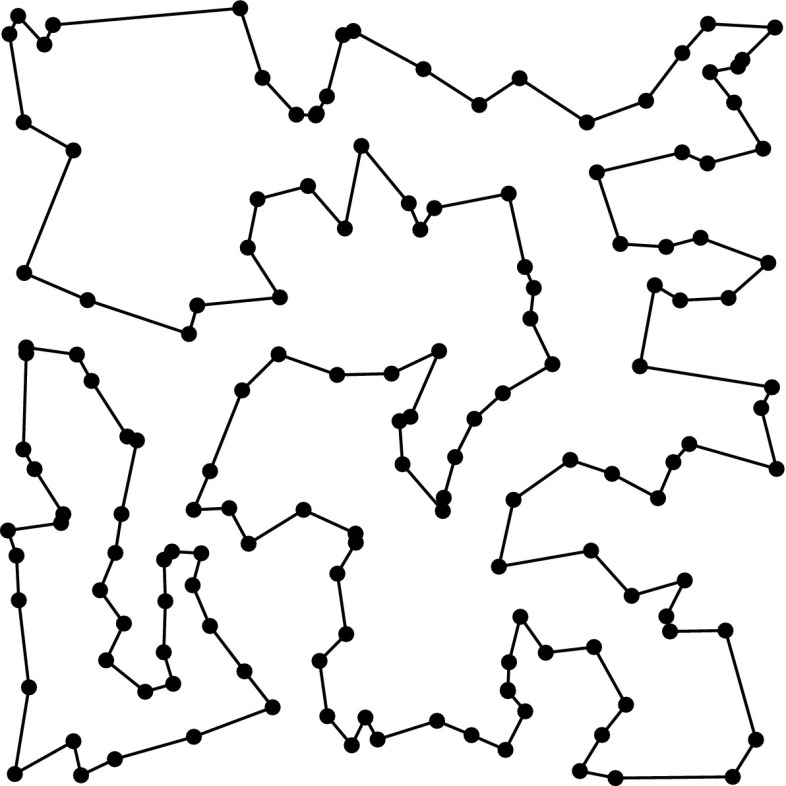
*RE_A_150*: iteration 9

**Fig. 16:**
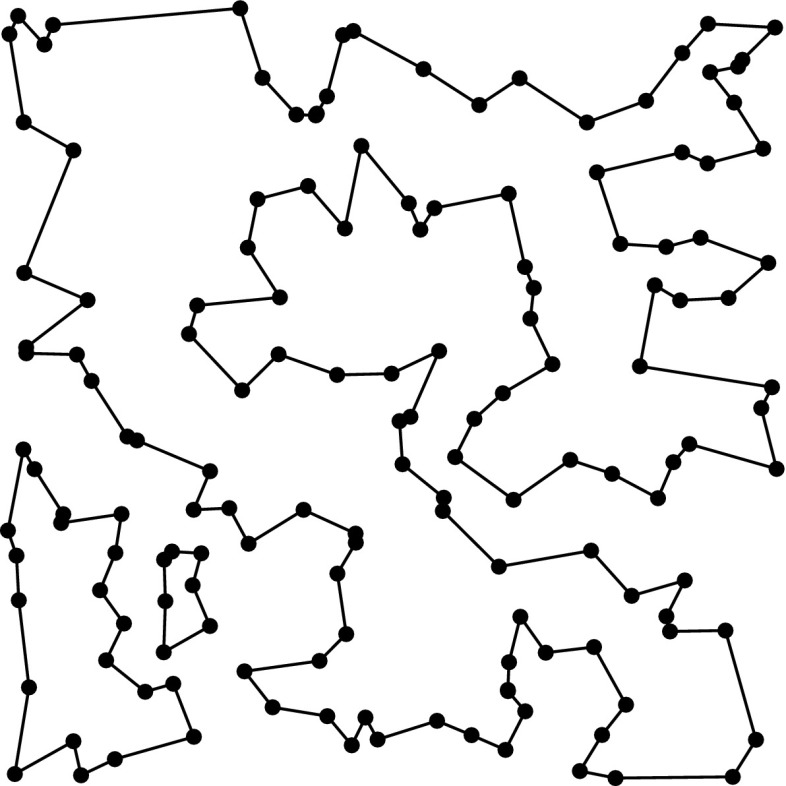
*RE_A_150*: iteration 10

**Fig. 17:**
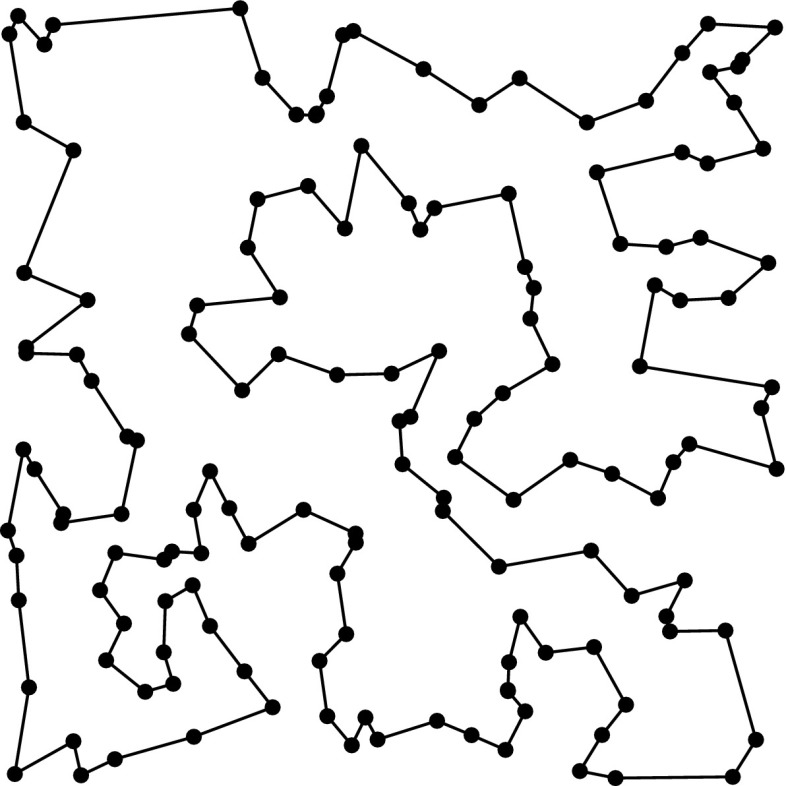
*RE_A_150*: iteration 11

**Fig. 18:**
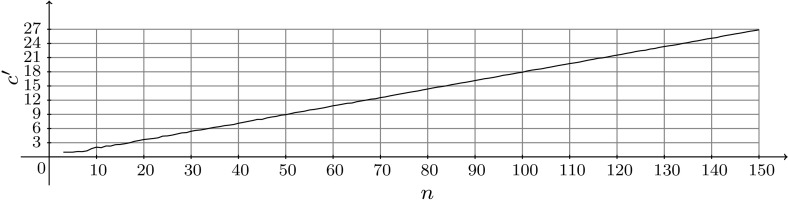
Restricted clustering with $c = n$ on random Euclidean graphs with minimum cluster size 3. The number of obtained clusters $c^\prime$ is plotted for every *n*. For every number of vertices *n* we created 100,000 graphs

We selected instances *kroB150* and *u159* whose vertices are depicted in Figs. 4 and 5 in the “Appendix”. Both instances consist of points in the Euclidean plane and the distances between every two vertices correspond to their respective Euclidean distances, however, they represent two very different instance types: The instance *kroB150* consists of relatively uniformly distributed points, the instance *u159* is more structured and it contains e.g. mesh substructures which are the worst setting for our algorithm (recall Sect. 4.1).

**Fig. 19:**
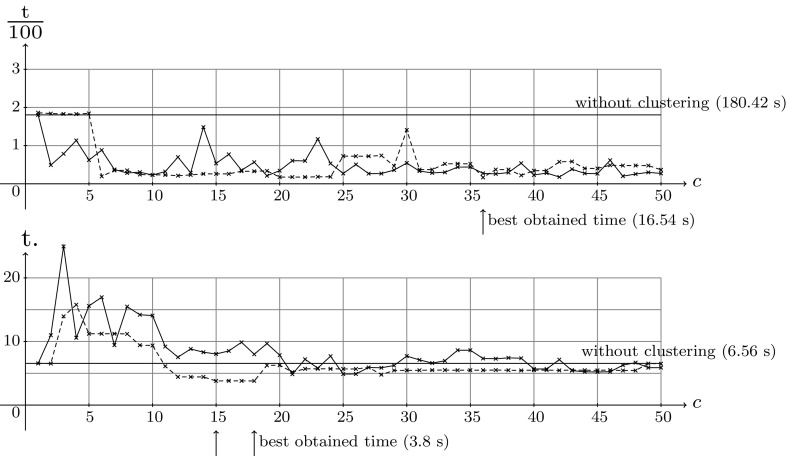
Computation time *t* in seconds depending on the number of clusters *c* for clustering (*full line*) and for restricted clustering (*dashed*). Illustrative instances *kroB150* (*upper figure*) and *u159* (*lower figure*)

Figure 19 in the “Appendix” illustrates the behavior of the running time *t* in seconds as a function of the parameter *c* for the instances *kroB150* and *u159*. The full lines correspond to standard clustering approach $C {\mid } c$ described in Sect. 3.2 (see Algorithm 2), while the dashed line corresponds to the restricted clustering $RC_3 {\mid } c$ of Sect. 3.3 with minimum cluster size 3. The standard BasicIntegerTSP without clustering arises for $c=1$.

Instance *kroB150* consists of relatively uniformly distributed points in the Euclidean plane, but has a specific property: By using Algorithm 2 we can observe the occurrence of two main components also for relatively small coefficient *c* (already for $c = 6$). This behavior is rather atypical for random Euclidean graphs, cf. (Penrose 2003, ch. 13), but it provides an advantage for our approach since we do not have to solve cluster instances of the same order of magnitude as the original graph but have several clusters of moderate size also for small cluster numbers *c*.

Considering the standard clustering approach (Algorithm 2) in Fig. 19, upper graph, it can be seen that only a small improvement occurs for *c* between 2 and 5. Looking at the corresponding clusterings in detail, it turns out in these cases that there exists only one “giant connected component” and all other clusters have size 1. This structure also implies that for the restricted clustering these isolated vertices are merged with the giant component and the effect of clustering is lost completely. For larger cluster numbers *c*, a considerable speedup is obtained, with some variation, but more or less in the same range for almost all values of $c\ge 6$ (in fact, the giant component splits in these cases). Moreover, the restricted clustering performs roughly as good as the standard clustering for $c \ge 6$.

Instance *u159* is much more structured and has many colinear vertices. Here, we can observe a different behavior. While the standard clustering is actually beaten by BasicIntegerTSP for smaller cluster numbers and has a more or less similar performance for larger cluster numbers, the restricted clustering is almost consistently better than the other two approaches. For *c* between 2 and 10 there exists a large component containing many mesh substructures which consumes as much computation time as the whole instance.

These two instances give some indication of how to characterize “good” instances for our algorithm: They should

- Consist of more clearly separated clusters and
- Not contain mesh substructures and colinear vertices.

### General computational results

A summary of the computational results for BasicIntegerTSP and the most promising variants of clustering based subtour generations can be found in Table 6. For random Euclidean instances we report only the mean values of all five instances of the same size [detailed results for all random Euclidean instances can be found in our accompanying technical report, Pferschy and Staněk (2015)]. It turns out that $\textit{HCD} {\mid } 4 \frac{n}{\log _2{n}}$, i.e. the hierarchical clustering approach combined with dropping SEC and fixing them only if they are generated again in the subsequent iteration and with the upper bound on the maximum cluster size $u = 4 \frac{n}{\log _2{n}}$, gives the best overall performance. A different behavior can be observed for instances taken from the TSPLIB and for random Euclidean instances. On the TSPLIB instances this algorithm $\textit{HCD} {\mid } 4 \frac{n}{\log _2{n}}$ is on average about 20 % faster than pure BasicIntegerTSP and beats the other clustering based approaches for most instances. In those cases, where it is not the best choice, it is usually not far behind.

As already mentioned, best results are obtained with $\textit{HCD} {\mid } 4 \frac{n}{\log _2{n}}$ for instances with a strong cluster structure and without mesh substructures (e.g. *pr299*). For instances with mesh substructures it is difficult to find an optimal 2-matching which is also a TSP tour. For random Euclidean instances the results are less clear but approaches with fixed number of clusters seem to be better then the hierarchical ones.

It was a main goal of this study to find a large number of “good” SEC, i.e. subtours that are present in the last iteration of the ILP-model of BasicIntegerTSP. Therefore, we show the potentials and limitations of our approach in reaching this goal. In particular, we will report the relation between the set $S_1$ consisting of all subtours generated by running a hierarchical clustering algorithm with an upper bound *u* (set as in the computational tests to $u = 4 \frac{n}{\log _2{n}}$) before solving the original problem (i.e. the root vertex) and the set $S_2$ containing only the SEC included in the final ILP model of BasicIntegerTSP. We tested the hierarchical clustering with and without the dropping of non-repeated subtours.

There are two aspects we want to describe: At first, we want to check whether $S_1$ contains a relevant proportion of “useful” subtour contraints, i.e. constraints also included in $S_2$, or whether $S_1$ contains “mostly useless” subtours. Therefore, we report the *proportion of used subtours* defined as

7 $$\begin{aligned} p_{used} :=\frac{\left| S_1\cap S_2\right| }{\left| S_1\right| }. \end{aligned}$$

Secondly, we want to find out to what extend it is possible to find the “right” subtours by our approach. Hence, we define the *proportion of covered subtours* defined as

8 $$\begin{aligned} p_{cov} :=\frac{\left| S_1 \cap S_2\right| }{\left| S_2\right| }. \end{aligned}$$

The values of $p_{used}$ and $p_{cov}$ are given in Table 3. It can be seen that empirically there is the chance to find about 26–31% ($p_{cov}$) of all required violated SEC. If SEC are allowed to be dropped, we are able to find fewer such constraints, but our choice has a better quality ($p_{cov}$ is smaller, but $p_{used}$ is larger), i.e. the solver does not have to work with a large number of constraints which only slow down the solving process and are not necessary to reach an optimal solution.

**Table 3:** Proportion of used and proportion of covered subtours for our hierarchical clustering approaches with the upper bound $u = 4 \frac{n}{\log _2{n}}$ which (i) does not allow ($\textit{HC} {\mid } 4 \frac{n}{\log _2{n}}$) and which (ii) does allow ($\textit{HCD} {\mid } 4 \frac{n}{\log _2{n}}$) to drop the unused SEC

Instance	$\textit{HC} {\mid } 4 \frac{n}{\log _2{n}}$		$\textit{HCD} {\mid } 4 \frac{n}{\log _2{n}}$	
	$p_{used}$	$p_{cov}$	$p_{used}$	$p_{cov}$
kroA150	0.26	0.46	0.48	0.37
kroB150	0.22	0.35	0.40	0.27
u159	0.09	0.45	0.15	0.39
brg180	0.13	0.15	0.71	0.15
kroA200	0.21	0.32	0.45	0.27
kroB200	0.21	0.41	0.42	0.39
tsp225	0.13	0.22	0.30	0.20
a280	0.06	0.31	0.16	0.28
lin318	0.23	0.38	0.44	0.36
gr431	0.07	0.21	0.22	0.19
pcb442	0.06	0.15	0.13	0.16
gr666	0.08	0.27	0.22	0.24
Mean	0.15	0.31	0.34	0.27
RE_A_150	0.18	0.31	0.29	0.24
RE_A_200	0.12	0.24	0.21	0.19
RE_A_250	0.12	0.27	0.17	0.20
RE_A_300	0.19	0.33	0.33	0.26
RE_A_350	0.15	0.38	0.29	0.33
RE_A_400	0.17	0.30	0.25	0.24
RE_A_450	0.15	0.42	0.31	0.37
RE_A_500	0.17	0.32	0.28	0.27
Mean	0.16	0.32	0.27	0.26
Mean of all	0.15	0.31	0.31	0.27

Furthermore, we can observe a relative big difference between the values of the proportion of used SEC ($p_{used}$) for the TSPLIB instances and for random Euclidean instances if the dropping of redundant constraints is allowed.

### Adding a starting heuristic

Of course, there are many possibilities of adding improvements to our basic approach. Lower bounds and heuristics can be introduced, branching rules can be specified, or cutting planes can be generated. We did not pursue these possibilities since we want to focus on the simplicity of the approach. Moreover, we wanted to take the ILP solver as a “black box” and not interfere with its execution.

Just as an example which immediately comes to mind, we added a starting heuristic to give a reasonably good TSP solution as a starting solution to the ILP solver. We used the improved version of the classical Lin–Kernighan heuristic in the code written by Helsgaun (2008). The computational results reported in Table 4 show that a considerable speedup (roughly a factor of 3, but also much more) can be obtained in this way.

**Table 4:** Results for BasicIntegerTSP used without/with the Lin–Kernighan heuristic for generating an initial solution: Using the starting heuristic yields faster running times for all instances

Instance	Without starting heuristic			With starting heuristic		
	t (s)	#iter	#SEC	t (s)	#iter	#SEC
kroA150	19	7	136	16	10	34
kroB150	179	8	148	17	8	104
u159	6	4	49	4	5	40
brg180	44	4	103	0	2	15
kroA200	677	8	237	42	8	135
kroB200	31	5	121	28	6	124
tsp225	178	9	261	73	13	176
a280	157	11	143	32	8	58
lin318	6885	8	357	4941	8	259
gr431	2239	9	453	838	10	318
pcb442	2737	11	501	447	18	207
gr666	17,711	8	789	13,225	11	485
Mean ratio				0.43	1.14	0.59
RE_A_150	23	8	100	14	11	65
RE_A_200	72	7	163	38	11	99
RE_A_250	138	9	186	63	9	124
RE_A_300	866	6	295	146	8	173
RE_A_350	411	5	252	126	6	151
RE_A_400	8456	8	454	1274	6	251
RE_A_450	2107	5	279	482	7	197
RE_A_500	15,330	6	436	1997	9	241
Mean ratio				0.32	1.27	0.62
Mean ratio all				0.39	1.19	0.60

*Mean ratios* refer to the arithmetic means over ratios between t (s)/#iter./#SEC for the approach using the starting heuristic over t (s)/#iter./#SEC for the BasicIntegerTSP. “t (s)” is the time in seconds, “#iter” the number of iterations and “#SEC” the number of SEC added to the ILP before starting the last iteration

## Some theoretical results and further empirical observations

Although our work mainly aims at computational experiments, we also tried to analyze BasicIntegerTSP from a theoretical point of view. In particular we studied the expected behavior on random Euclidean instances and tried to characterize the expected cardinality of the minimal set of required subtours $\mathcal {S}^*$ as defined in Sect. 2. It is well known that no polynomially bounded representation of the TSP polytope can be found and there also exist instances based on a mesh-structure for which $\mathbb {E}\left[ \left| S^*\right| \right]$ has exponential size, but the question for the expected size of $\left| S^*\right|$ for random Euclidean instances and thus for the expected number of iterations of our solution algorithm remains an interesting open problem.

**Fig. 20:**
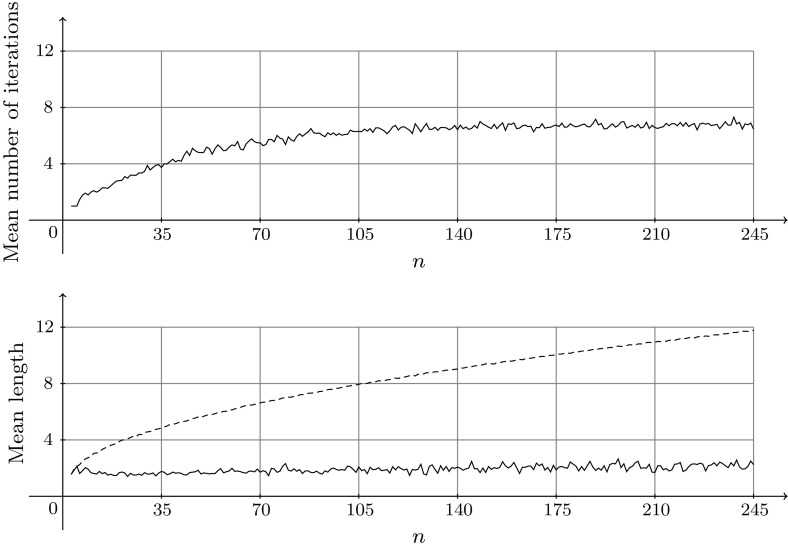
Mean number of iterations used by the BasicIntegerTSP (*upper figure*), mean length of an optimal TSP tour (*lower figure*, *dashed*) and mean length of an optimal weighted 2-matching (*lower figure*, *full line*) in random Euclidean graphs. For every number of vertices *n* we created 100 graphs

**Fig. 21:**
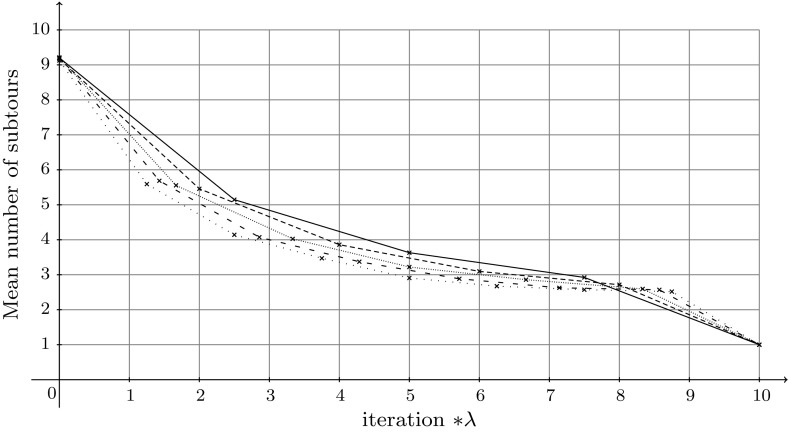
Mean number of subtours during the BasicIntegerTSP in random Euclidean graphs for $n = 60$ sorted according to the number of iterations [$\lambda = 4/10$ (*full line*), 5/10 (*dashed*), 6/10 (*dotted*), 7/10 (*loosely dashed*), 8/10 (*loosely dotted*)]. We created 100,000 graphs

We started with extensive computational tests, some of them presented in Figs. 20 and 21 in the “Appendix”, to gain empirical evidence on this aspect. The upper graph in Fig. 20 illustrates the mean number of iterations needed by BasicIntegerTSP to reach optimality for different numbers of vertices *n* (we evaluated 100 random Euclidean instances for every value *n*). The lower graph of Fig. 20 shows the mean length of the optimal TSP tour and of the optimal 2-matching (i.e. the objective value after solving the ILP in the first iteration) by using the same setting.

It was proven by Beardwood et al. (1959) that the expected length of an optimal TSP tour is asymptotically $\beta \sqrt{n}$, where $\beta$ is a constant. This approach was later generalized for other settings and other properties of the square root asymptotic were identified by Rhee (1993) and Yukich (1998). We used these properties to prove the square root asymptotic also for the 2-matching problem (cf. Fig. 20, lower graph, dashed).

### **Definition 1**

[*complete convergence,* Yukich (1998)] A sequence of random variables $X_n$, $n \ge 1$, *converges completely (c.c.)* to a constant *C* if and only if for all $\varepsilon > 0$ we have

9 $$\begin{aligned} \sum _{n = 1}^{\infty }{\mathbb {P}\left[ |X_n - C| > \varepsilon \right] } < \infty . \end{aligned}$$

### **Theorem 1**

Let $G = (V, E)$ be a random Euclidean graph with $n = |V|$ vertices and let $d :E \rightarrow \mathbb {R}^+_0$ be the Euclidean distance function. Furthermore, let $M_2(G, d)$ be the length of an optimal 2-matching. Then

10 $$\begin{aligned} \lim _{n \rightarrow \infty } \frac{M_2(G, d)}{\sqrt{n}} = \alpha \ \hbox { c.c., where } \alpha > 0. \end{aligned}$$

### *Proof*

See our accompanying technical report, Pferschy and Staněk (2015). $\square$

Based on these results the following idea might lead to a proof that the expected cardinality $\mathcal {S}^*$ is polynomially bounded: After the first iteration of the algorithm we have a solution possibly consisting of several separate subtours of total asymptotic length $\alpha \sqrt{n} = \alpha _1 \sqrt{n}$. If there are subtours, we add SEC (in fact at most $\lfloor \frac{n}{3}\rfloor$), resolve the enlarged ILP and get another solution whose asymptotic length is $\alpha _2 \sqrt{n}$. By proving that the expected length of the sequence $\alpha = \alpha _1, \ldots , \alpha _{\#i} = \beta$ is polynomially bounded in *n*, one would obtain that also $\mathbb {E}\left[ \left| S^*\right| \right]$ is polynomially bounded since only polynomially many subtours are added in each iteration. Our intuition and computational tests illustrated in Fig. 20, upper graph, indicate that the length of this sequence could be proportional to $\sqrt{n}$ as well. Unfortunately, we could not find the suitable techniques to show this step.

A different approach is illustrated in Fig. 21, where we examine the mean number of subtours contained in every iteration. In particular, we chose $n = 60$, generated 100,000 random Euclidean instances and sorted them by the number of iterations #iter required by BasicIntegerTSP. The most frequent number of ILP solver runs was 7 (dotted line), but we summarize the results for 5 (full line), 6 (dashed), 8 (loosely dashed) and 9 (loosely dotted) necessary runs in this figure as well. For every iteration of every class (with respect to the number of involved ILP runs) we compute the mean number of subtours contained in the respective solutions. As can be expected these numbers of subtours are decreasing (in average) over the number of iterations. To allow a better comparison of this behavior for different numbers of iterations we scaled the iteration numbers into the interval [0, 10] (horizontal axis of Fig. 21). It can be seen that the average number of subtours contained in an optimal 2-matching (first iteration) is about 9.2 while in the last iteration we trivially have only one tour. Between these endpoints we can first observe a mostly convex behavior, only in the last step before reaching the optimal TSP tour a sudden drop occurs. It would be interesting to derive an asymptotic description of these curves. An intuitive guess would point to an exponential function, but so far we could not find a theoretical justification of this claim.

## Conclusions

In this paper we provide a “test of concept” of a very simple approach to solve TSP instances of medium size to optimality by exploiting the power of current ILP solvers. The approach consists of iteratively solving ILP models with relaxed SEC to integer optimality. Then it is easy to find integral subtours and add the corresponding SEC to the ILP model. Iterating this process until no more subtours are contained in the solution obviously solves the TSP to optimality. It would also be possible to treat subtour elimination constraints as so-called *lazy cuts* and invoke them only when a new integer solution is found. However, since the number of generated constraints remains moderate in our test instances we found no advantage in pursuing this option.

In this work we focus on the structure of SEC and how to find a “good” set of SEC in reasonable time. Therefore, we aim to identify the local structure of the vertices of a given TSP instance by running a clustering algorithm. Based on empirical observations and results from random graph theory we further extend this clustering-based approach and develop a hierarchical clustering method with a mechanism to identify SEC as “relevant”, if they appear in consecutive iterations of the algorithm.

We mostly refrained from adding additional features which are highly likely to improve the performance considerably, such as starting heuristics (cf. Sect. 4.4), lower bounds or adding additional cuts. In the future it might be interesting to explore the limits of performance one can reach with a purely integer linear programming approach by adding these improvements. Clearly, we can not expect such a basic approach to compete with the performance of *Concorde*, Applegate et al. (2006), which has been developed over many years and basically includes all theoretical and technical developments known so far. However, it turns out that most of the standard benchmark instances with up to 400 vertices can be solved in a few minutes by this purely integer strategy.

Finally, we briefly discussed some theoretical aspect for random Euclidean graphs which could lead to polyhedral results in the expected case.

## Appendix

**Table 5:** Results for BasicIntegerTSP and for different variants of the approach which uses the hierarchical clustering

Instance	BasicIntegerTSP			$\textit{HC} {\mid } n$			$\textit{HC} {\mid } 4 n / \log _2{n}$			$\textit{HCD} {\mid } 4 n / \log _2{n}$		
	t (s)	#iter	#SEC	t (s)	#iter	#SEC	t (s)	#iter	#SEC	t (s)	#iter	#SEC
ch150	13	7	74	150	2	435	**9**	**5**	**223**	14	6	129
kroA150	19	7	136	11	2	268	**8**	**2**	**245**	11	4	130
kroB150	179	8	148	72	2	301	34	4	315	**21**	**4**	**168**
pr152	16	13	184	**5**	**3**	**214**	**5**	**3**	**205**	9	4	174
u159	**6**	**4**	**49**	29	3	292	14	4	303	11	4	140
si175	52	10	183	99	6	494	**40**	**8**	**415**	44	7	263
brg180	44	4	103	54	2	185	102	18	359	**24**	**2**	**27**
rat195	347	6	274	**241**	**3**	**491**	272	4	419	267	5	322
d198	10,986	10	301	**483**	**7**	**894**	1094	10	582	3986	9	326
kroA200	677	8	237	**177**	**2**	**362**	941	3	353	690	5	238
kroB200	31	5	121	37	3	292	**23**	**3**	**269**	31	4	164
gr202	**39**	**11**	**77**	2430	3	1216	61	8	330	60	8	217
tsp225	178	9	261	1113	3	981	**138**	**6**	**551**	151	6	341
pr226	5183	10	409	18	1	593	**13**	**1**	**585**	59	3	357
gr229	239	6	311	2984	4	1056	**172**	**7**	**490**	173	8	324
gil262	179	7	268	1052	2	807	**169**	**3**	**564**	217	4	368
a280	157	11	143	–	–	–	**124**	**3**	**733**	181	7	352
pr299	9263	9	413	4051	2	782	1998	5	745	**1716**	**5**	**455**
lin318	6885	8	357	457	2	756	**274**	**5**	**660**	275	5	355
rd400	2401	9	467	9329	5	1494	**983**	**6**	**1018**	1579	8	539
gr431	**2239**	**9**	**453**	–	–	–	4748	9	1734	4214	10	833
pcb442	2737	11	501	–	–	–	3830	16	1796	**2277**	**15**	**888**
u574	17,354	6	423	–	–	–	18,050	4	1290	**8664**	**4**	**629**
gr666	**17,711**	**8**	**789**	–	–	–	23,212	4	3104	18,031	7	1408
rat783	**30,156**	**6**	**457**	–	–	–	–	–	–	–	–	–
Mean ratio				6.02	0.40	3.41	0.89	0.81	2.67	0.80	0.75	1.42
Mean RE_150	**12.4**	**6.6**	**72.6**	69.8	2	327	17	3.8	231.8	14.6	4.4	133.4
Mean RE_200	75	7.2	146	340.6	2	510.8	**51.4**	**4.2**	**340.4**	94	4.4	223
Mean RE_250	262.4	6.6	208.8	845.6	2.2	717	**135**	**4.8**	**472**	227.2	5	302.4
Mean RE_300	830.8	7	308.8	2780.8	3	947.4	**408.2**	**5.2**	**591.4**	557.4	5.6	398.6
Mean RE_350	790.8	6.6	294.2	5015.6	2.4	1047.4	**562.4**	**4.2**	**696.8**	581.8	4.6	413.2
Mean RE_400	20,596.8	7.2	397.4	304,882.4	2.4	1630.2	14,541.4	4.8	834.6	**10,422.2**	**4.6**	**516.4**
Mean RE_450	23,911.2	7.4	409.4	–	–	–	**19,705.8**	**5**	**988.8**	22,828.8	5.6	598
Mean RE_500	120,453.2	7	492.4	–	–	–	**98,447.6**	**4.6**	**1172.4**	106,721.2	6	714.6
Mean ratio				12.13	0.34	3.80	0.90	0.67	2.47	1.04	0.73	1.50
Mean ratio all				9.76	0.37	3.65	0.90	0.72	2.54	0.95	0.74	1.47

*Mean ratios* refer to the arithmetic means over ratios between t (s)/#iter./#SEC for the particular approaches and t (s)/#iter./#SEC for the BasicIntegerTSP. “t (s)” is the time in seconds, “#iter” the number of iterations and “#SEC” the number of SEC added to the ILP before starting the last iteration. The entries “–” for TSPLIB instances cannot be computed with 16 GB RAM or would take more than 12 hours

**BasicIntegerTSP**

${{\mathbf {HC}}} {\mid } {{\mathbf {n}}}$—hierarchical clustering; the constraints cannot be dropped and the maximum size of a solved cluster is $u = n$ (i.e. in fact, there is no upper bound)

${{\mathbf {HC}}} {\mid } \mathbf{4} {{\mathbf {n}}}/ {\mathbf{log}}_{\mathbf{2}}{{{\mathbf {n}}}}$—hierarchical clustering; the constraints cannot be dropped and the maximum size of a solved cluster is $u = 4 \frac{n}{\log _2{n}}$

${{\mathbf {HCD}}} {\mid } \mathbf{4} {{\mathbf {n}}} / {\mathbf{log}}_\mathbf{2}{{{\mathbf {n}}}}$—hierarchical clustering; the constraints can be dropped and the maximum size of a solved cluster is $u = 4 \frac{n}{\log _2{n}}$

The data for the best approach with respect to running time is given in bold

**Table 6:** Comparison between different variants of our approach

Instance	BasicIntegerTSP			$C {\mid } \lfloor n / 5 \rfloor$			$RC_3 {\mid } \lfloor n / 5 \rfloor$			$RC_3 {\mid } n$			$\textit{HCD} {\mid } 4 n / \log _2{n}$		
	t (s)	#iter	#SEC	t (s)	#iter	#SEC	t (s)	#iter	#SEC	t (s)	#iter	#SEC	t (s)	#iter	#SEC
ch150	13	7	74	12	6	114	**9**	**6**	**109**	16	7	117	14	6	129
kroA150	19	7	136	25	6	187	43	6	166	33	5	185	**11**	**4**	**130**
kroB150	179	8	148	53	4	219	138	5	215	44	5	202	**21**	**4**	**168**
pr152	16	13	184	17	12	181	17	11	204	18	12	181	**9**	**4**	**174**
u159	6	4	49	6	4	149	5	5	151	**3**	**3**	**70**	11	4	140
si175	52	10	183	**31**	**10**	**213**	55	13	250	35	9	196	44	7	263
brg180	44	4	103	**17**	**3**	**81**	19	8	102	121	11	316	24	2	27
rat195	347	6	274	246	4	268	275	6	315	**114**	**6**	**257**	267	5	322
d198	10,986	10	301	4253	11	315	–	–	–	4762	9	321	**3986**	**9**	**326**
kroA200	677	8	237	332	6	214	350	5	190	**287**	**4**	**171**	690	5	238
kroB200	31	5	121	29	5	148	**21**	**5**	**147**	32	4	123	31	4	164
gr202	39	11	77	50	8	233	36	6	174	**25**	**6**	**143**	60	8	217
tsp225	178	9	261	100	9	223	**84**	**10**	**235**	100	8	300	151	6	341
pr226	5183	10	409	3614	6	363	36,744	5	403	12,944	9	415	**59**	**3**	**357**
gr229	239	6	311	335	6	289	**152**	**6**	**256**	311	7	341	173	8	324
gil262	179	7	268	250	8	250	**133**	**7**	**268**	152	6	274	217	4	368
a280	157	11	143	**61**	**4**	**299**	196	11	350	117	9	221	181	7	352
pr299	9263	9	413	6376	7	387	7410	6	416	16059	6	414	**1716**	**5**	**455**
lin318	6885	8	357	537	7	331	386	6	364	1560	6	391	**275**	**5**	**355**
rd400	2401	9	467	**1212**	**7**	**420**	1827	7	438	1522	8	398	1579	8	539
gr431	**2239**	**9**	**453**	3098	9	626	3384	9	647	2496	10	704	4214	10	833
pcb442	2737	11	501	3868	16	770	**1815**	**17**	**567**	2626	16	594	2277	15	888
u574	17,354	6	423	11,702	4	498	35,204	5	580	13,722	5	572	**8664**	**4**	**629**
gr666	17,711	8	789	**11,756**	**7**	**919**	14,223	7	1001	13,573	7	1002	18,031	7	1408
rat783	**30,156**	**6**	**457**	184,381	5	701	37,805	5	735	38,630	6	779	–	–	–
Mean ratio				1.01	0.86	1.31	1.17	0.95	1.34	0.98	0.94	1.29	0.80	0.75	1.42
Mean RE_150	12.4	6.6	72.6	**9.6**	**4.6**	**105**	12.6	4.8	116.2	13.6	4.8	104.4	14.6	4.4	133.4
Mean RE_200	75	7.2	146	55.6	5.6	207.4	**44.6**	**5.4**	**185.6**	56.6	5.6	184.2	94	4.4	223
Mean RE_250	262.4	6.6	208.8	**164.4**	**5.8**	**239.4**	287	6	283.2	226.6	6	248.8	227.2	5	302.4
Mean RE_300	830.8	7	308.8	721.8	6.4	353.8	525.6	6.2	339.2	586.2	6.2	340.6	557.4	5.6	398.6
Mean RE_350	790.8	6.6	294.2	580.8	6.2	322.2	655.4	5.8	333.6	**540.8**	**5.2**	**339.6**	581.8	4.6	413.2
Mean RE_400	20,596.8	7.2	397.4	18,619.4	6	453.4	21,482.4	5.8	448.4	18,520	6.6	465.2	**10,422.2**	**4.6**	**516.4**
Mean RE_450	23,911.2	7.4	409.4	12119.2	6.8	481.8	11,018.4	5.8	478.6	**10,649**	**6.6**	**468.8**	22,828.8	5.6	598
Mean RE_500	120,453.2	7	492.4	141,573.2	6.6	593.4	**78,874.8**	**5.6**	**560**	118,289.6	6.8	572.2	106,721.2	6	714.6
Mean ratio				0.90	0.87	1.24	0.96	0.83	1.27	1.18	0.87	1.23	1.04	0.73	1.50
Mean ratio all				0.94	0.87	1.27	1.04	0.87	1.29	1.10	0.90	1.25	0.95	0.74	1.47

*Mean ratios* refer to the arithmetic means over ratios between t (s)/#iter./#SEC for the particular approaches and t (s)/#iter./#SEC for the BasicIntegerTSP. “t (s)” is the time in seconds, “#iter” the number of iterations and “#SEC” the number of SEC added to the ILP before starting the last iteration. The entries “–” for TSPLIB instances cannot be computed with 16 GB RAM

**BasicIntegerTSP**

$${{\mathbf {C}}} {\mid } \lfloor {{\mathbf {n}}} / \mathbf{5} \rfloor $$—clustering for $$c = \lfloor \frac{n}{5}\rfloor $$

${{\mathbf {RC}}}_{\mathbf{3}} {\mid } \lfloor {{\mathbf {n}}} / {{\mathbf {5}}} \rfloor$—restricted clustering for $c = \lfloor \frac{n}{5}\rfloor$; the minimum size of a cluster is 3

${{\mathbf {RC}}}_{\mathbf{3}} {\mid } {{\mathbf {n}}}$—restricted clustering for $c = n$; the minimum size of a cluster is 3

${{\mathbf {HCD}}}{\mid } \mathbf{4} {{\mathbf {n}}} / {\mathbf{log}}_{\mathbf{2}}{{{\mathbf {n}}}}$—hierarchical clustering; the constraints can be dropped and the maximum size of a solved cluster is $u = 4 \frac{n}{\log _2{n}}$

The data for the best approach with respect to running time is given in bold

## Footnotes

1 Precise version: *Linux 3.8.0-29-generic #42~precise1-Ubuntu SMP x86_64 x86_64 x86_64 GNU/Linux*

2 Precise compiler version: *gcc version 4.6.3*.

3 Precise version: *SCIP version 3.0.1 [precision: 8 byte] [memory: block] [mode: optimized] [LP solver: CPLEX 12.4.0.0] [GitHash: 9ee94b7] Copyright (c) 2002–2013 Konrad-Zuse-Zentrum für Informationstechnik Berlin (ZIB)*.

4 We represented all distances as integers by scaling with $2^{14}$ and rounding to the nearest integer.
